# Laparoscopic Bilateral Nerve‐Sparing Retroperitoneal Lymph Node Dissection for Testicular Cancer After Chemotherapy

**DOI:** 10.1111/iju.70267

**Published:** 2025-10-28

**Authors:** Koji Hatano, Yu Ishizuya, Yuichiro Nakamura, Masataka Kawamura, Shigeaki Nakazawa, Norichika Ueda, Takuji Hayashi, Yoshiyuki Yamamoto, Kentaro Takezawa, Kensuke Mitsunari, Taigo Kato, Yoichi Kakuta, Atsunari Kawashima, Shinichiro Fukuhara, Ryoichi Imamura, Norio Nonomura

**Affiliations:** ^1^ Department of Urology The University of Osaka Graduate School of Medicine Suita Japan; ^2^ Department of Urology Nagasaki University Graduate School of Biomedical Sciences Nagasaki Japan; ^3^ Department of Urology Japan Community Health Care Organization Osaka Hospital Osaka Japan

**Keywords:** laparoscopic surgery, lymph node dissection, lymphatic metastasis, nerve‐sparing, testicular cancer

## Abstract

**Objectives:**

Nerve‐sparing techniques are commonly used in retroperitoneal lymph node dissection (RPLND) for patients with testicular cancer to preserve postoperative ejaculation. Laparoscopic RPLND is applicable to small residual masses; however, nerve‐sparing techniques remain challenging. This study aimed to establish a safe, bilateral nerve‐sparing laparoscopic RPLND via the transperitoneal approach. The technique was developed through cadaveric surgical training (CST) and applied clinically.

**Methods:**

Thiel‐fixed human cadavers were used for CST. In patients with testicular cancer after chemotherapy, laparoscopic RPLND was performed based on normalized tumor markers, typically with a residual mass of 5 cm or less. Patients were placed in the lateral position, and four to six ports were used, including the umbilical port; central ports were shared bilaterally. The dissection extent followed a bilateral full‐template approach.

**Results:**

In CST, lumbar splanchnic nerves were identified and preserved, supporting the clinical feasibility of laparoscopic RPLND. The bilateral nerve‐sparing laparoscopic RPLND was performed in 10 patients: 6 with non‐seminoma and 4 with seminoma. The mean pre‐chemotherapy tumor diameter was 2.7 cm (range, 1.2–5.6 cm). The mean blood loss was 165 mL (range, 0–540 mL). The bilateral lumbar splanchnic nerves were preserved in all cases, and postoperative ejaculatory function was confirmed to be preserved in 9 cases. Surgical complications included Grade 1 chyle leakage in 5 patients, all of whom improved with conservative management.

**Conclusions:**

A laparoscopic bilateral nerve‐sparing RPLND technique was developed to achieve both minimal invasiveness and preservation of ejaculatory function. Long‐term follow‐up is necessary to confirm oncological outcomes.

## 1 Introduction

Testicular cancer is most commonly diagnosed in males aged 15 to 40 years, with approximately 2.5 cases per 100 000 individuals annually in Japan [1]. Although chemotherapy is effective for testicular cancer, even in advanced stages, survivors may experience infertility, sexual dysfunction, and other adverse effects. The primary challenge in treating advanced cases lies in improving curability while minimizing these risks. After chemotherapy for metastatic testicular cancer, surgical resection of residual tumors is recommended if tumor markers have normalized, and additional chemotherapy may be considered if viable tumor cells are identified. Retroperitoneal lymph node dissection (RPLND) is a key procedure, as retroperitoneal lymph nodes are the most frequent site of metastasis.

The standard approach for post‐chemotherapy RPLND involves a full template, using the renal vein, ureters, and common iliac arteries as anatomical landmarks. Nerve‐sparing techniques have been developed to preserve ejaculatory function by protecting the sympathetic trunk, lumbar splanchnic nerves, and hypogastric plexus [2]. At Osaka University, Nonomura et al. previously reported that 22 (84.6%) of 26 Japanese men with advanced testicular cancer who underwent nerve‐sparing RPLND after chemotherapy achieved antegrade ejaculation [3]. Anatomic studies have shown that retroperitoneal lymph node metastases are typically located ipsilateral to the testicular tumor and below the renal vein, prompting the development of modified dissection templates. These modified templates reduce contralateral dissection to preserve nerves related to ejaculation; however, they may fail to detect residual cancer in 3%–32% of cases [4, 5]. Therefore, bilateral full‐template RPLND with nerve‐sparing techniques may offer effective oncologic control while preserving quality of life.

Laparoscopic RPLND is typically indicated for small tumors. In 2001, LeBlanc et al. introduced a retroperitoneal lateral approach for RPLND, which was generally performed using a modified template [6]. In Japan, laparoscopic RPLND via this approach has been reported in post‐chemotherapy patients with testicular cancer [7, 8, 9]. However, performing bilateral templates with nerve‐sparing techniques remains challenging. Additionally, this approach has drawbacks, such as difficulty in maintaining a clear surgical field if the peritoneum is injured. In 2008, Steiner et al. reported a nerve‐sparing RPLND via a transperitoneal approach, which was considered feasible by experienced surgeons [10]. To date, laparoscopic RPLND with nerve‐sparing has not yet been widely adopted as a standard procedure.

This study aimed to establish a safe, bilateral nerve‐sparing, full‐template laparoscopic RPLND via the transperitoneal approach. The technique was developed through cadaveric surgical training (CST) and subsequently applied in clinical practice.

## 2 Methods

### 2.1 CST

This study was approved by the institutional review board of Osaka University Hospital [No. 18394(T1)‐9, 98], and written informed consent was obtained prior to death in all cases. Thiel‐fixed human cadavers were used to perform laparoscopic RPLND in CST. Cadavers were placed in the lateral position, and an EZ access port (Hakko Medical, Chikuma, Japan) was inserted at the umbilical region. Left‐ and right‐sided laparoscopic RPLND were performed separately, each using four ports, including the umbilical port. The AirSeal system (CONMED, Largo, FL) was used for CO_2_ insufflation at a pressure of 10 mmHg. A LigaSure vessel‐sealing device (Covidien Japan, Tokyo, Japan) was used for tissue sealing.

### 2.2 Patients

Laparoscopic RPLND has been performed since March 2023. Patients were included based on normalization of tumor markers following cisplatin‐based chemotherapy. Laparoscopic RPLND was typically performed approximately 6 weeks after chemotherapy to evaluate for residual tumor or teratoma. The primary indication for laparoscopic RPLND was clinical stage IIA or IIB disease, with a pre‐chemotherapy mass of 5 cm or less and limited encasement of the inferior vena cava and/or aorta. However, if the operating surgeon determined that the procedure was feasible, it was performed regardless of tumor size or disease stage. This procedure was approved by the institutional review board of Osaka University Hospital as an advanced new medical technology and was subsequently conducted under insurance coverage by the Japanese government. The use of clinical data and intraoperative imaging was also approved by the institutional review board of Osaka University Hospital (No. 13397‐23). All patients were fully informed and provided written consent for both the surgery and the use of their clinical data. Postoperative ejaculatory function was assessed and recorded by inquiring about the presence or absence of ejaculation during outpatient visits.

### 2.3 Surgical Procedure

Patients were placed in the lateral position, and an EZ access port was inserted at the umbilical region. Laparoscopic bilateral nerve‐sparing RPLND via the transperitoneal approach was initiated on the left side with the patient in the right lateral position, followed by the right side in the left lateral position. The reason for starting from the left side is to ensure sufficient space around the aorta, making it easier to identify the aorta from the right side. Typically, four to six ports were used, including the umbilical port, with the central ports shared bilaterally. Additional ports were permitted if maintaining the surgical field became difficult. The insufflation pressure was set at 10 mmHg using the AirSeal system.

The main surgical procedures for bilateral nerve‐sparing RPLND were as follows: (1) exposure of the great vessels using retroperitoneal maneuvers; (2) dissection of the inferior vena cava and aorta using the split‐and‐roll technique; (3) identification and preservation of the ureter, renal artery, inferior mesenteric artery, and common iliac artery; (4) identification and preservation of the sympathetic trunks and lumbar splanchnic nerves; (5) tumor resection and lymph node dissection while exposing the great vessels and preserving the nerves; and (6) removal of the gonadal vein on the affected side. The hypogastric plexus was identified by following the lumbar splanchnic nerves distally and was preserved as pre‐aortic tissue below the inferior mesenteric artery. The lumbar veins were transected as needed to allow mobilization of the inferior vena cava. Bilateral nerve‐sparing was the standard approach; however, the nerve fibers to be preserved were selected according to the tumor's size, location, and degree of adhesion. When dissecting around nerves, cold dissection using scissors or dissecting forceps was employed as the basic technique, avoiding the use of energy devices. Extensive sealing of the lymphatic vessels was performed using LigaSure along all dissection borders. For tumors adherent to the inferior vena cava or adjacent organs, the tip of the dissecting forceps was kept slightly away from the vessel wall to prevent inadvertent vascular injury, and dissection was performed using the curvature of the forceps.

All surgical procedures were performed by KH.

## 3 Results

### 3.1 CST

The cadavers were placed in the lateral position. Left‐ and right‐sided laparoscopic RPLND were performed separately. On the left side, the descending colon and spleen were mobilized to expose the aorta. The left sympathetic trunk was identified posterior to the left renal vein. The left lumbar splanchnic nerve, which arises from the sympathetic trunk, could be preserved (Figure S1a). On the right side, the ascending colon and duodenum were mobilized to expose the inferior vena cava and the aorta. The right lumbar splanchnic nerves were identified in the aortocaval region and could also be preserved (Figure S1b). Laparoscopic surgery with an umbilical port was determined to be clinically applicable for bilateral nerve‐sparing RPLND.

### 3.2 Patient Characteristics

Laparoscopic bilateral nerve‐sparing RPLND was performed in 10 patients with testicular cancer following chemotherapy (Table 1). Histological types included six cases of non‐seminoma and four cases of seminoma. The primary sites of testicular cancer were the right testis in five cases and the left testis in five cases. The main sites of retroperitoneal lymph node metastasis were the para‐aortic region in six cases and the aortocaval region in four cases. The mean tumor diameter was 2.7 cm (range, 1.2–5.6 cm) before chemotherapy and 1.0 cm (range, 0.5–1.8 cm) before surgery. Case 10 represented a late recurrence following chemotherapy in a patient who had not previously undergone RPLND.

**TABLE 1:** Patient characteristics.

Case	Age	Laterality of testis cancers	TNM classification	Clinical stage	Histological type	IGCCC risk	Main LN metastatic sites	Tumor size before chemotherapy (cm)	Chemotherapy	Tumor size before surgery (cm)
1	38	Lt	pT1N1M0S1	IIA	EC, seminoma and YST	Good	Para‐aortic	1.3	BEP × 3	0.9
2	30	Lt	pT2N2M0S1	IIB	EC and seminoma	Good	Para‐aortic	3	BEP × 3	1.1
3	35	Rt	pT2N2M0S0	IIB	Seminoma	Good	Aortocaval	3.6	BEP × 4	1.6
4	50	Rt	pT1N1M0S1	IIA	Seminoma	Good	Aortocaval	1.5	EP × 4	0.5
5	38	Lt	pT2N3M0S0	IIC	Seminoma	Good	Para‐aortic	5.6	BEP × 3	1.8
6	36	Rt	pT1N1M0S1	IIA	EC, seminoma and teratoma	Good	Aortocaval	1.9	BEP × 3	0.7
7	31	Lt	pT3N2M1bS3	IIIC	YST and teratoma	Poor	Para‐aortic	4.7	BEP × 4, TIP× 1, IrN × 4	0.8
8	46	Lt	pT2N1M0S1	IIA	Seminoma	Good	Para‐aortic	1.6	EP × 4	0.5
9	18	Rt	pT2N1M0S1	IIA	Choriocarcinoma	Good	Para‐aortic	1.2	BEP × 3	1
10	49	Rt	pTxNxM1aSx	III	EC and YST	Unknown	Aortocaval	Unknown	BEP × 4	1.4

Abbreviations: BEP, bleomycin, etoposide and cisplatin; EC, embryonal carcinoma; EP, etoposide and cisplatin; IGCCC, International Germ Cell Classification Consensus; IrN, irinotecan and nedaplatin; LN, lymph node; Lt, left; Rt, right; TIP, paclitaxel, ifosfamide and cisplatin; YST, yolk sac tumor.

### 3.3 Surgical Outcomes

**FIGURE 1:**
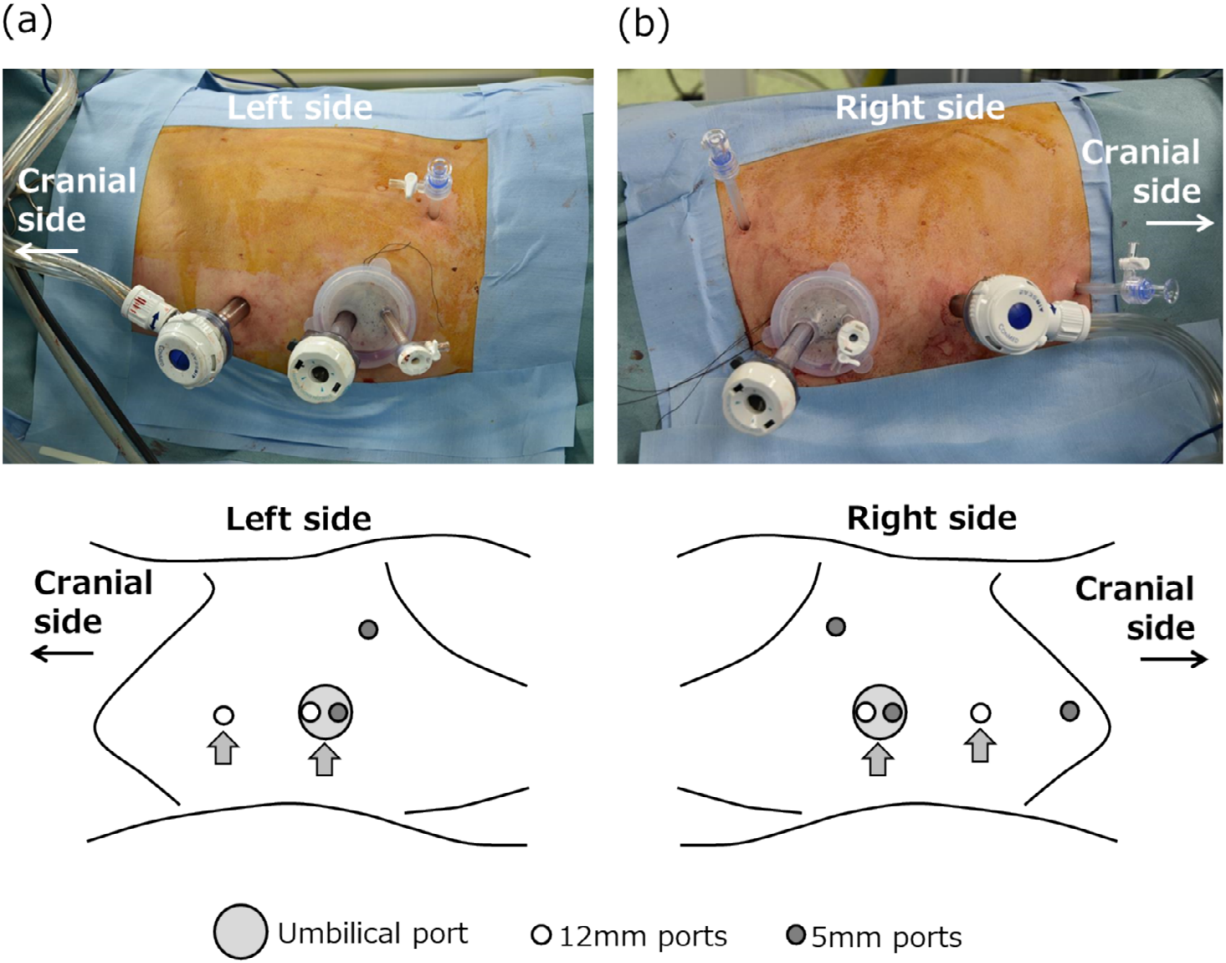
Port arrangement for laparoscopic bilateral nerve‐sparing RPLND in Case 3. (a) Left‐sided dissection with the patient in the right lateral position; (b) Right‐sided dissection with the patient in the left lateral position. Five ports were used, including the umbilical port. Arrows indicate central ports shared bilaterally.

A typical example of port arrangement is shown in Figure 1. This configuration was considered satisfactory from a cosmetic standpoint (Figure S2). Typically, four to six ports were used, including the umbilical port; however, two patients with a body mass index greater than 30 required additional ports.

**FIGURE 2:**
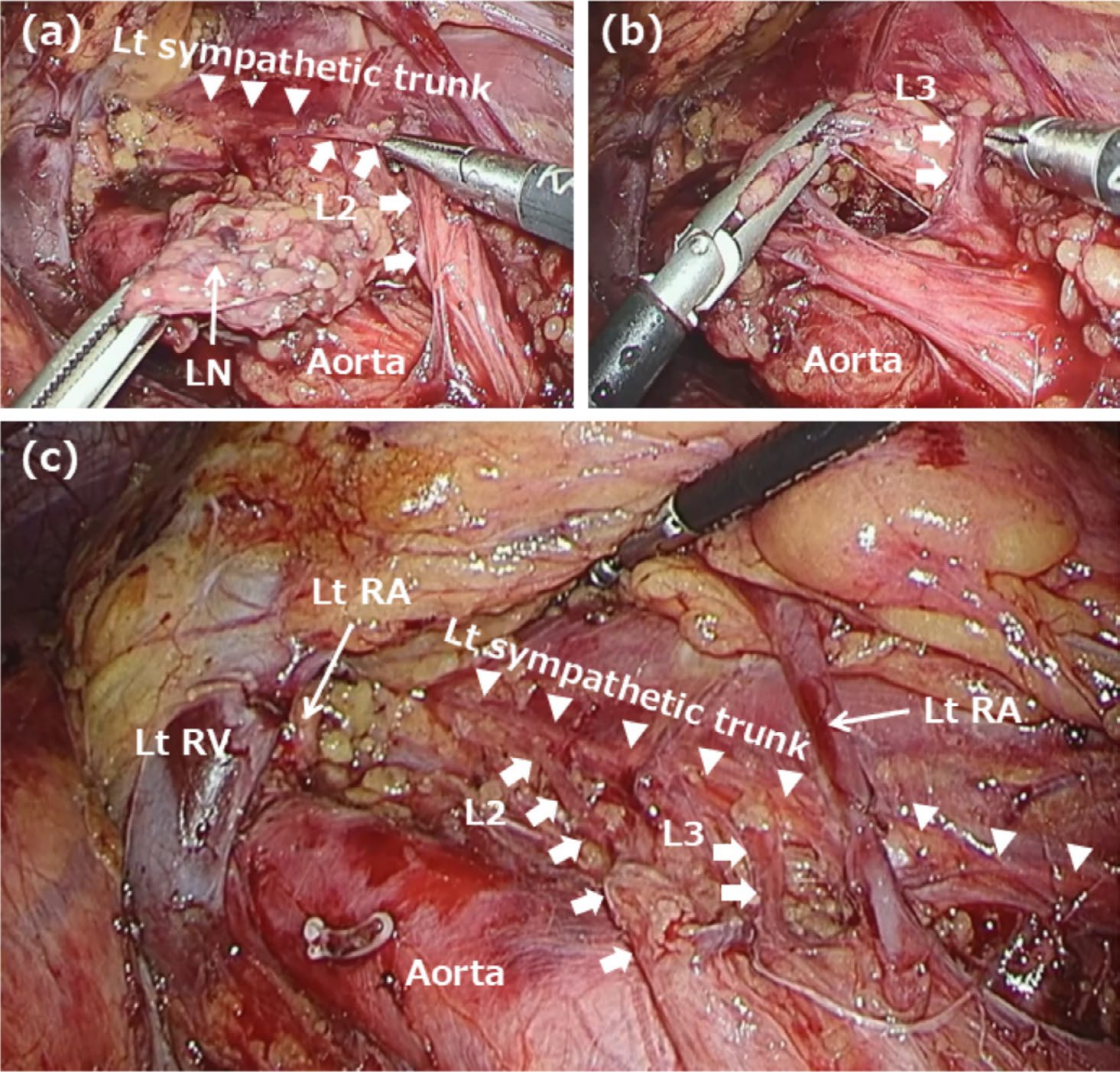
Intraoperative findings of laparoscopic left‐sided nerve‐sparing RPLND in Case 6. (a) The left sympathetic trunk was identified extending along the iliopsoas muscle. The L2 lumbar splanchnic nerve was preserved during para‐aortic lymph node dissection. (b) The L3 lumbar splanchnic nerve was also preserved. (c) Findings after lymph node dissection. L, lumbar splanchnic nerve; LN, lymph node; Lt, left; RA, renal artery; RV, renal vein.

**FIGURE 3:**
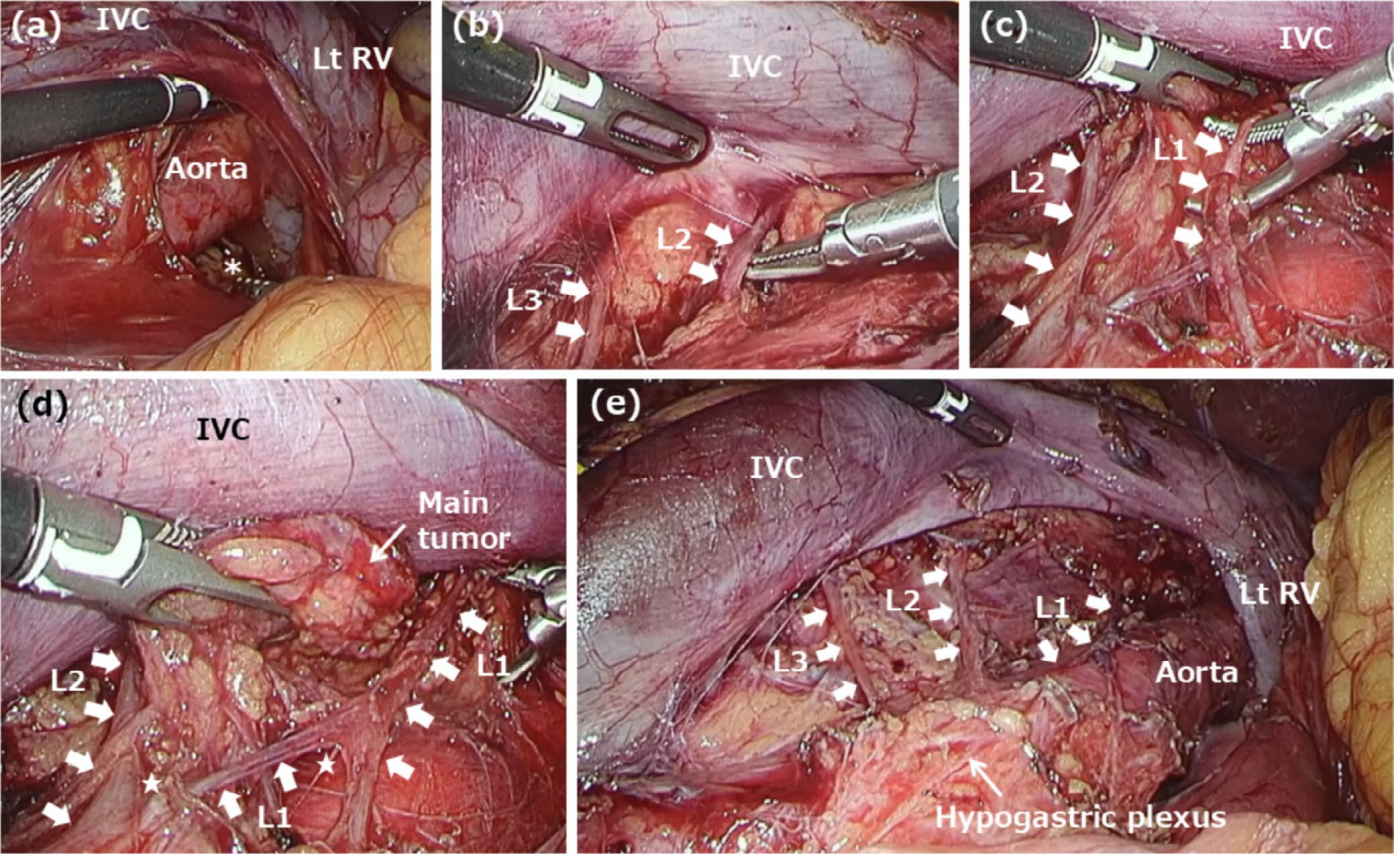
Intraoperative findings of laparoscopic right‐sided nerve‐sparing RPLND in Case 6. (a) Following exposure of the inferior vena cava and aorta, the left‐sided dissection area was visualized (asterisk). (b, c) The right lumbar splanchnic nerves were identified in the aortocaval region. (d) The main tumor was dissected while preserving the lumbar splanchnic nerves. Branching and fused nerve fibers were observed (star). (e) Findings after lymph node dissection. The hypogastric plexus was preserved. IVC, inferior vena cava; L, lumbar splanchnic nerve; Lt, left; RV, renal vein.

**FIGURE 4:**
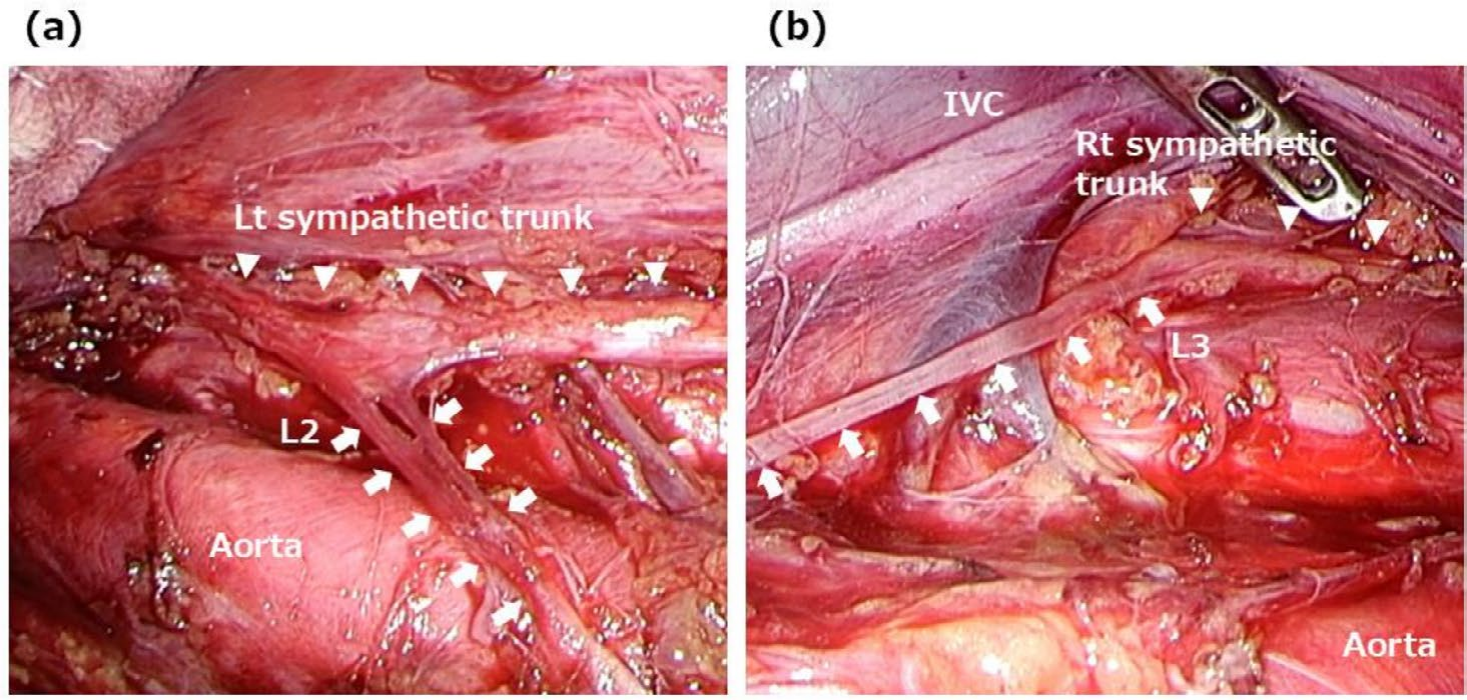
Intraoperative findings after RPLND in Case 7. Bilateral lumbar splanchnic nerves arising from the sympathetic trunks were visualized on both sides: (a) left side; (b) Right side. IVC, inferior vena cava; L, lumbar splanchnic nerve; Lt, left; Rt, right.

Intraoperative findings are illustrated using Case 6 as a representative example. On the left side, the descending colon and spleen were mobilized to expose the renal hilum and aorta (Figure 2). The left sympathetic trunk was identified posterior to the left renal vessels, extending along the iliopsoas muscle. The left lumbar splanchnic nerves, which arise from the sympathetic trunk, were preserved during para‐aortic lymph node dissection. On the right side, the ascending colon and duodenum were mobilized to expose the inferior vena cava and aorta, connecting to the previously dissected area on the left (Figure 3). The right lumbar splanchnic nerves were identified in the aortocaval region after the inferior vena cava was dissected using the split‐and‐roll technique. The right lumbar splanchnic nerves and the contiguous hypogastric plexus were preserved during dissection of the main tumor and aortocaval lymph nodes. Laparoscopic views allowed for identification of nerve fiber branching and fusion. Bilateral lumbar splanchnic nerves arising from the sympathetic trunks were clearly visualized in other cases, such as Case 7 (Figure 4) and Case 9 (Figure S3), using the magnified view of the laparoscope.

The summary of surgical outcomes for the 10 cases is presented in Table 2. The mean operative time was 248 min (range, 154–429 min) for the right side and 234 min (range, 108–329 min) for the left side. The mean estimated blood loss was 165 mL (range, 0–540 mL), and no patients required blood transfusion. No cases required conversion to open surgery. Several nerve fibers were resected due to tumor adhesion, but the bilateral lumbar splanchnic nerves were preserved in all cases. Postoperative ejaculatory function was confirmed to be preserved in nine cases at a median of 1 month (range 1–5 months). Surgical complications included Grade 1 chyle leakage in five patients, all of whom improved with conservative management. Lymph node pathology revealed necrosis in six cases, viable cancer in three cases, and teratoma in one case. Among these, in case 9 with right testicular cancer, viable cancer was detected in the para‐aortic lymph node, which was outside the modified template [5]. In patients with viable cancer, two additional courses of chemotherapy were administered to achieve disease‐free status. No recurrence was observed during a median follow‐up of 8 months (range, 1–28 months).

**TABLE 2:** Surgical outcomes.

Case	Surgery time Rt (min)	Surgery time Lt (min)	Blood loss (mL)	Preserved lumbar splanchnic nerves	Postoperative ejaculatory function	Pathology of lymph nodes	Location of viable cancer or teratoma	Complications (Grade)
1	257	264	50	Rt L1‐3, and Lt L1‐3	Yes	Viable cancer	Para‐aortic	Chyle leakage (Grade 1)
2	159	228	27	Rt L1‐4, and Lt L3	Yes	Necrosis	N/A	None
3	429	108	180	Rt L2‐3, and Lt L1‐3	Yes	Necrosis	N/A	Chyle leakage (Grade 1)
4	362	241	240	Rt L1‐3, and Lt L2‐3	Yes	Necrosis	N/A	None
5	179	240	110	Rt L2‐4, and Lt L2‐3	No	Necrosis	N/A	Chyle leakage (Grade 1)
6	234	227	20	Rt L1‐3, and Lt L2‐3	Yes	Teratoma	Aortocaval	Chyle leakage (Grade 1)
7	154	315	0	Rt L1, 3, and Lt L2‐3	Yes	Necrosis	N/A	None
8	176	175	210	Rt L1‐3, and Lt L1‐3	Yes	Necrosis	N/A	Chyle leakage (Grade 1)
9	207	329	275	Rt L1‐3, and Lt L1‐3	Yes	Viable cancer	Para‐aortic	None
10	321	212	540	Rt L2‐3, and Lt L1‐3	Yes	Viable cancer	Aortocaval	None

Abbreviations: Lt, left; N/A, not applicable; Rt, right.

## 4 Discussion

In this study, bilateral nerve‐sparing laparoscopic RPLND was successfully established via the transperitoneal approach. The surgical technique was developed through CST and safely implemented in clinical practice. The magnified view provided by the laparoscope enabled clear visualization of the lumbar splanchnic nerves, which facilitated their preservation, during RPLND for small residual masses. Laparoscopic RPLND was minimally invasive and provided favorable cosmetic outcomes.

In this study, we applied the CST using Thiel‐embalmed human cadavers to develop the laparoscopic RPLND technique. CST with Thiel cadavers is well suited for establishing advanced laparoscopic procedures, as it closely replicates tissue color, consistency, and operative tactility [11]. Bilateral nerve‐sparing RPLND aims to preserve ejaculatory function; however, this is technically challenging due to the complex neurovascular anatomy. Cadaveric studies enhance surgeons' understanding of the lumbar splanchnic nerves and support preservation of ejaculatory function after surgery [12]. Beveridge et al. demonstrated that the position of the lumbar splanchnic nerves varies significantly between specimens [13]. Therefore, the magnified view of the laparoscope is advantageous for nerve identification and preservation in CST, making it well suited for clinical training.

Neuroanatomical studies of the retroperitoneum have indicated that preservation of the sympathetic trunk, lumbar splanchnic nerves from L1 to L3, and the hypogastric plexus is necessary to maintain regulated antegrade ejaculation [5, 14]. Because the sympathetic trunk and lumbar splanchnic nerves are bilateral, unilateral preservation has been considered sufficient to maintain normal ejaculatory function. To confirm the effectiveness of nerve‐sparing, Kaiho et al. performed electrostimulation of the lumbar splanchnic nerves associated with ejaculation during RPLND and monitored seminal emission using endoscopic visualization of the posterior urethra to observe the response [15]. Electrostimulation of the lumbar splanchnic nerves elicited only ipsilateral seminal emission, suggesting that some efferent sympathetic signals for emission may travel ipsilaterally in humans [15]. Miki et al. reviewed 78 cases of nerve‐sparing RPLND and reported that antegrade ejaculation was preserved in 93.3% of bilateral nerve‐sparing cases and in 74.3% of unilateral nerve‐sparing cases [16]. Therefore, although bilateral nerve‐sparing is desirable, when tumors are large or involve adhesions, the nerve fibers to be preserved should be selected on a case‐by‐case basis.

Currently, RPLND after chemotherapy for testicular cancer is generally performed via open surgery, while laparoscopic surgery may be indicated by specialists in selected cases. Laparoscopic RPLND is typically indicated when tumor markers have normalized following chemotherapy, the pre‐chemotherapy tumor size is less than 5 cm, and there is limited invasion of the inferior vena cava or aorta [8, 17]. In this study, patient selection adhered to these criteria. Laparoscopic RPLND has demonstrated outcomes comparable to open surgery in selected cases; however, modified template resections have been more frequently performed than radical bilateral resections [18]. Arai et al. reported that extraperitoneal laparoscopic RPLND can be performed with acceptable morbidity and excellent oncological control in patients with testicular cancer following chemotherapy [8]. In their cohort, modified templates were applied in most cases. Therefore, bilateral templates with nerve‐sparing techniques remain technically challenging in laparoscopic RPLND. Kimura et al. employed a nerve‐sparing technique for extraperitoneal laparoscopic RPLND after chemotherapy [19], and Nakamura et al. reported that extraperitoneal nerve‐sparing laparoscopic RPLND using bilateral templates was safe and effective, with a high preservation rate of antegrade ejaculation (92.9%) [9]. In the present study, patients with relatively small residual masses were selected for transperitoneal laparoscopic nerve‐sparing RPLND. Some nerve fibers were resected due to tumor adhesions; however, bilateral nerve sparing was achieved in all cases. Notably, antegrade ejaculation was confirmed to be preserved in 9 out of 10 cases. The magnified view of the laparoscope allowed clear visualization of the lumbar splanchnic nerves, which was useful for understanding the trajectories of nerve fibers and for educating junior physicians. Furthermore, in one of the 10 cases in this study, viable cancer was detected in a lymph node outside the modified template [5]. Therefore, in selected cases, bilateral templates may contribute to cancer control.

This study has several limitations. First, due to the short observation period, it was not possible to evaluate the oncological outcomes of this procedure. Atypical recurrence following laparoscopic or robotic RPLND has been reported [20, 21], and long‐term outcome data remain limited. Second, the sample size in this study was small; therefore, further case accumulation is warranted. In addition, chyle leakage was identified as a common complication of this procedure. In this study, chyle leakage occurred in five out of ten patients; however, all cases were Grade 1 and resolved with conservative management. Notably, extraperitoneal laparoscopic RPLND is also associated with a relatively high incidence of chyle and/or lymphatic leakage [8, 9, 19]. This study included cases of seminoma with small residual tumors, but no viable cancer was detected following RPLND. Following cisplatin‐based chemotherapy for advanced seminoma, viable cancers were detected in 12%–30% of men with residual tumors greater than 3 cm and in less than 10% of men with residual tumors less than 3 cm [22, 23]. Therefore, observation could be an option for seminoma cases with residual tumors less than 3 cm.

Although not yet approved in Japan, robot‐assisted RPLND may become the standard approach in the future [24, 25, 26]. Nerve‐sparing techniques have also been applied in robot‐assisted surgery [20]. Various patient positions and port placements have been investigated in robotic RPLND. In the present study, the umbilical port was utilized and may be adaptable to robotic single‐port surgery in the future.

In conclusion, we developed a laparoscopic, nerve‐sparing, full‐template RPLND technique that aims to achieve both minimal invasiveness and preservation of ejaculatory function. Long‐term follow‐up is necessary to confirm oncological outcomes.

## Author Contributions

**Koji Hatano:** conceptualization, data curation, formal analysis, funding acquisition, investigation, methodology, project administration, visualization, writing – original draft, writing – review and editing. **Yu Ishizuya:** data curation, writing – review and editing. **Yuichiro Nakamura:** data curation. **Masataka Kawamura:** data curation. **Shigeaki Nakazawa:** data curation. **Norichika Ueda:** data curation. **Takuji Hayashi:** data curation. **Yoshiyuki Yamamoto:** data curation. **Kentaro Takezawa:** data curation. **Kensuke Mitsunari:** data curation. **Taigo Kato:** data curation. **Yoichi Kakuta:** data curation. **Atsunari Kawashima:** data curation. **Shinichiro Fukuhara:** data curation. **Ryoichi Imamura:** data curation. **Norio Nonomura:** conceptualization, funding acquisition, project administration, supervision, writing – review and editing.

## Ethics Statement

This study was approved by the institutional review board of Osaka University Hospital [no. 18394(T1)‐9, 98, and no. 13397‐23].

## Consent

All participants were informed and provided written consent.

## Conflicts of Interest

Kentaro Takezawa and Norio Nonomura are the editorial board members of the International Journal of Urology and co‐authors of this article. To minimize bias, they were excluded from all editorial decision‐making related to the acceptance of this article for publication. All other authors declare no conflicts of interest.

## Supporting information


**Figure S1:** Cadaveric surgical training for (a) left‐sided and (b) right‐sided laparoscopic RPLND. Cadavers were placed in the lateral position, and left‐ and right‐sided laparoscopic RPLND were performed separately.
(a) The left sympathetic trunk and left lumbar splanchnic nerve were identified. The lumbar splanchnic nerve extended anterior to the aorta. (b) The right lumbar splanchnic nerves were identified in the aortocaval region and could be preserved. IVC, inferior vena cava; L, lumbar splanchnic nerve; Lt, left; RV, renal vein.
**Figure S2:** Postoperative appearance of laparoscopic RPLND in Case 3.
**Figure S3:** Intraoperative findings after RPLND in Case 9. Bilateral lumbar splanchnic nerves arising from the sympathetic trunks were visualized on both sides: (a) Left side; (b) Right side. IVC, inferior vena cava; L, lumbar splanchnic nerve; Lt, left; Rt, right; RV, renal vein.
