# Supplementary material for: Laparoscopic Bilateral Nerve‐Sparing Retroperitoneal Lymph Node Dissection for Testicular Cancer After Chemotherapy
Source: Int J Urol. 2025 Oct 28;33(1):e70267. doi: 10.1111/iju.70267 (PMC12835680; doi:10.1111/iju.70267)

**Supplementary Figure legends**

**Supplementary Figure 1.**

Cadaveric surgical training for (a) left-sided and (b) right-sided laparoscopic RPLND. Cadavers were placed in the lateral position, and left- and right-sided laparoscopic RPLND were performed separately.
(a) The left sympathetic trunk and left lumbar splanchnic nerve were identified. The lumbar splanchnic nerve extended anterior to the aorta. (b) The right lumbar splanchnic nerves were identified in the aortocaval region and could be preserved.
IVC, inferior vena cava; L, lumbar splanchnic nerve; Lt, left; RV, renal vein.

**Supplementary Figure 2.**

Postoperative appearance of laparoscopic RPLND in Case 3.

**Supplementary Figure 3.**

Intraoperative findings after RPLND in Case 9. Bilateral lumbar splanchnic nerves arising from the sympathetic trunks were visualized on both sides:
(a) Left side; (b) Right side.
IVC, inferior vena cava; L, lumbar splanchnic nerve; Lt, left; Rt, right; RV, renal vein.

**Supplementary Figure 1**

**
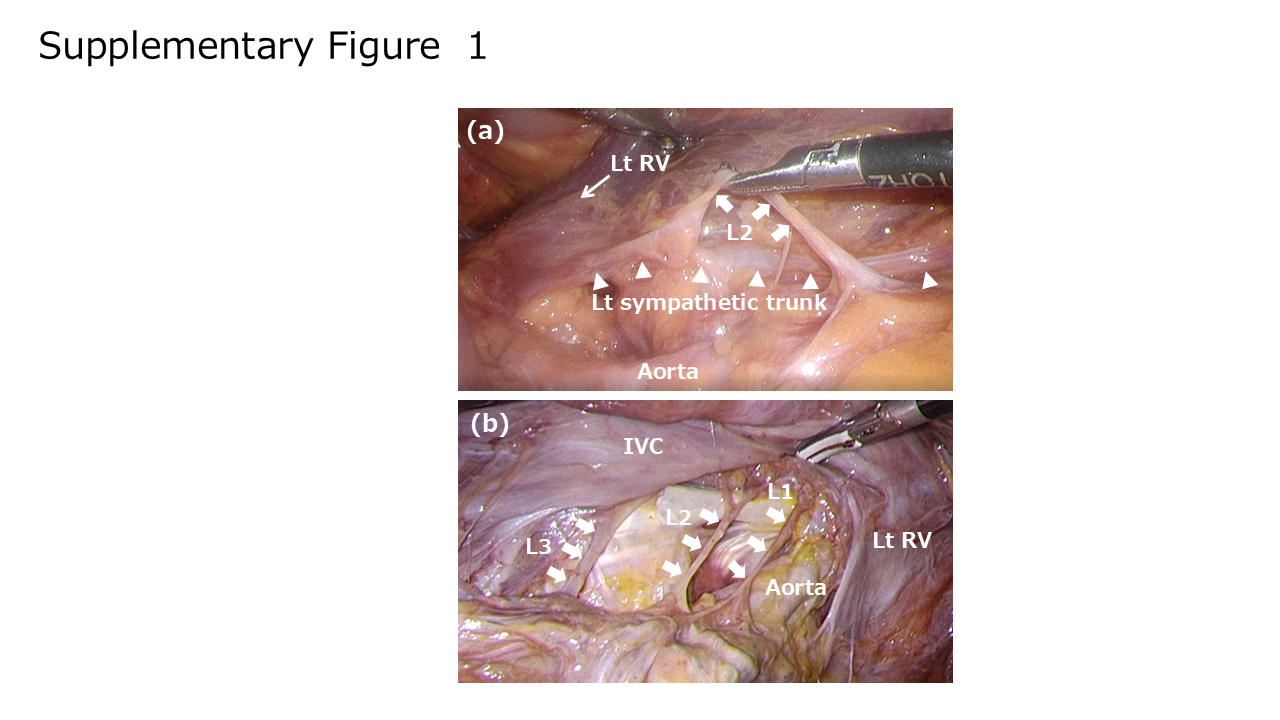
**

**Supplementary Figure 2**

**
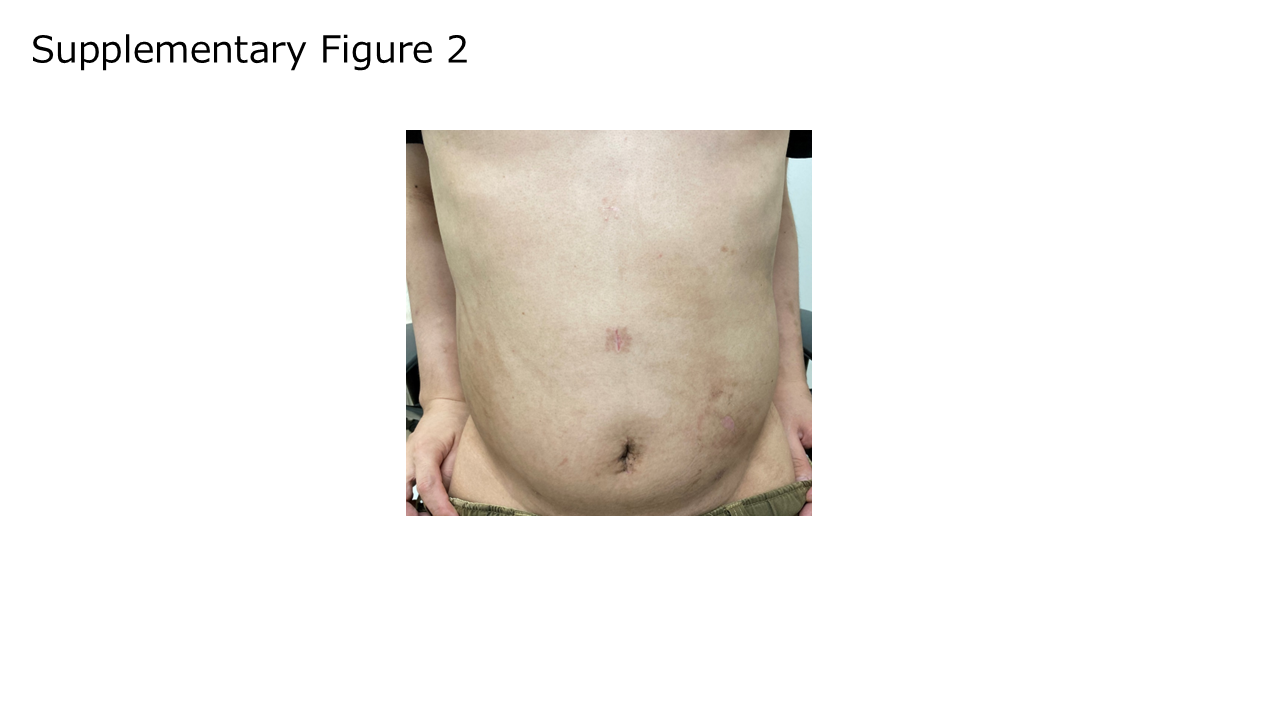
**

**Supplementary Figure 3**


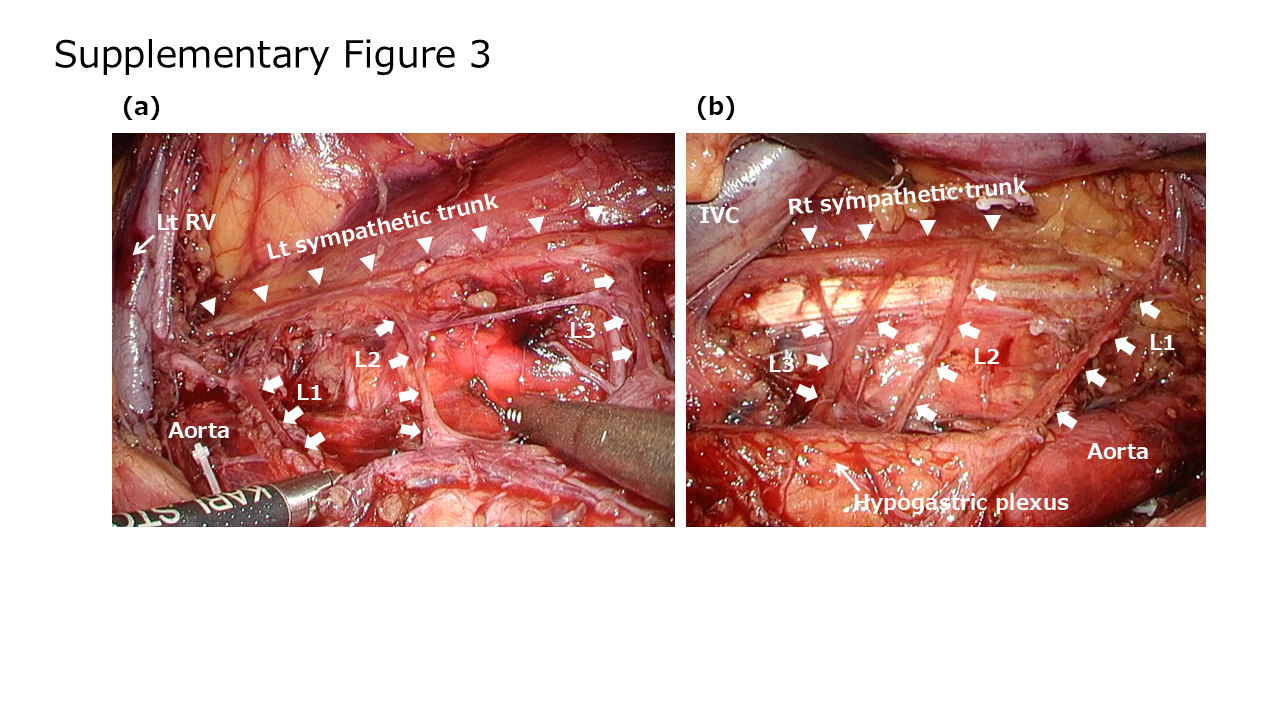

Supplement: Supplementary file 1 — Figure S1: Cadaveric surgical training for (a) left‐sided and (b) right‐sided laparoscopic RPLND. Cadavers were placed in the lateral position, and left‐ and right‐sided laparoscopic RPLND were performed separately. (a) The left sympathetic trunk and left lumbar splanchnic nerve were identified. The lumbar splanchnic nerve extended anterior to the aorta. (b) The right lumbar splanchnic nerves were identified in the aortocaval region and could be preserved. IVC, inferior vena cava; L, lumbar splanchnic nerve; Lt, left; RV, renal vein. Figure S2: Postoperative appearance of laparoscopic RPLND in Case 3. Figure S3: Intraoperative findings after RPLND in Case 9. Bilateral lumbar splanchnic nerves arising from the sympathetic trunks were visualized on both sides: (a) Left side; (b) Right side. IVC, inferior vena cava; L, lumbar splanchnic nerve; Lt, left; Rt, right; RV, renal vein. [file IJU-33-0-s001.docx]
